# Evaluation of the Acute and Sub-Acute Oral Toxicity of Jaranol in Kunming Mice

**DOI:** 10.3389/fphar.2022.903232

**Published:** 2022-06-30

**Authors:** Tianlong Liu, Yao Zhang, Jing Liu, Junwen Peng, Xin Jia, Yunfeng Xiao, Lanbing Zheng, Yu Dong

**Affiliations:** ^1^ Department of Pharmacy, Affiliated Hospital of Inner Mongolia Medical University, Hohhot, China; ^2^ College of Pharmacy, Inner Mongolia Medical University, Hohhot, China; ^3^ Department of Natural Medicinal Chemistry, College of Pharmacy, Inner Mongolia Medical University, Hohhot, China; ^4^ Engineering Technology Research Center of Pharmacodynamic Substance and Quality Control of Mongolian Medicine in Inner Mongolia, Hohhot, China; ^5^ Center for New Drug Safety Evaluation and Research, Inner Mongolia Medical University, Hohhot, China; ^6^ Department of Psychiatry, Inner Mongolia Mental Health Center, Hohhot, China

**Keywords:** jaranol, acute toxicity, sub-acute toxicity, mice, safety

## Abstract

**Background:** Jaranol has shown a wide range of pharmacological activities; however, no study has yet examined *in vivo* toxicity. The study aimed to investigate the oral acute and sub-acute toxicity of jaranol in mice.

**Methods:** The acute toxicity was determined by a single oral dose of jaranol (2000 mg/kg). Therein animal behaviour and mortality rate were observed for 14 days. The jaranol (50, 100 and 200 mg/kg BW·d^−1^) was given by gavage for 28 days daily in the sub-acute study. The mouse body weight (BW), organ weight, food, water intake, biochemical, haematological parameters, and histopathology were studied in acute and sub-acute toxicity.

**Results:** During the acute toxicity test, a single oral dose (2000 mg/kg) jaranol did not cause significant alteration in majority of the hematological indices. However, jaranol decreased the level of serum alanine aminotransferase and aspartate aminotransferase. Those results showed that the oral lethal dose 50 (LD_50_) of jaranol was higher than 2000 mg/kg BW, regardless of sex. In repeated daily oral doses (50, 100 and 200 mg/kg BW·d^−1^), no mortality was recorded in the various experimental groups. The jaranol reduced body weight gain (200 mg/kg BW·d^−1^), the relative spleen weight (all doses) and serum alanine aminotransferase activity (200 mg/kg BW·d^−1^). On the other hand, jaranol significantly elevated red blood cell count (100 and 200 mg/kg BW·d^−1^) and serum creatinine levels (200 mg/kg BW·d^−1^). Histological study revealed that spleen bleeding was identified in 200 mg/kg jaranol-treated mice.

**Conclusion:** Jaranol was relatively safe in Kunming Mice when repetitively administered orally in small doses for a prolonged period of time. We recommend more chronic toxicity studies and clinical trials on jaranol to ensure that its use is free of potential toxicity to humans.

## 1 Introduction

Traditional medicine still plays an important role in delaying, preventing and treating diseases worldwide, especially in developing countries (Yuan et al., 2016). Terpenoids, alkaloids, flavonoids, phenolics and other ingredients from medicinal herbs could regulate multiple human physiological functions by complex mechanisms. Among these active ingredients, plant-derived flavonoids, including isoflavones, are attracting growing interest due to their diverse biological functions. The plant-derived flavonoids are widely found in fruits, vegetables, nuts and medicinal herbs (Lin et al., 2021). On one hand, flavonoids are important active ingredients of multiple traditional Chinese medicines and have diverse biological functions, including anti-oxidant, anti-inflammatory and anti-carcinogenic effects (Perez-Vizcaino and Fraga 2018). On the other hand, it was reported that flavonoids have potential flavonoid/phenolic toxicity, including pro-oxidant activity and mitochondrial toxicity (Said Ahmad et al., 1992; Galati and O'Brien 2004). Previous studies showed that flavonoids were autoxidized in the presence of O_2_ and transition metals such as copper (Cu) and iron (Fe), which led to the generation of reactive oxygen species (ROS) and phenoxyl radicals that can damage DNA, lipids, and other biological molecules (Decker 1997). In addition, ROS and phenoxyl radicals from flavonoids are an important cause of mitochondrial dysfunction related to multiple human diseases (Babu et al., 2013; Agrawal and Jha 2020). The toxic effect of flavonoids has a certain significance in cancer therapy, but this also acts as an obstacle for their application in the treatment of other diseases.

Jaranol, a plant-derived flavonoid, could be extracted from various traditional medicinal herbs, such as *licorice (Glycyrrhiza* spp.*)*, *Psychotria serpens* and *Siparuna cristata* (Chen 2011; Xue et al., 2013). Some studies illustrated that jaranol had a potential role in inhibiting SARS-CoV-2 replication (Leal et al., 2021) and exerting anti-cancer (Woo et al., 2017), alpha-glucosidase inhibitory (Dao et al., 2021), anti-bacterial (Zhang et al., 2019), and anti-oxidant activities *in vitro* (Quek et al. 2021). At present, no studies evaluating the effect of jaranol *in vivo* were found from a PubMed search with the keywords jaranol or kumatakenin. To reveal jaranol candidate targets and mechanisms, a network pharmacology-based strategy was performed by our team. The results showed that nicotinamide adenine dinucleotide phosphate (NADPH) oxidase 4 (NOX4) was a putative target regulated by jaranol, while NOX4 played a critical role in the pathophysiology of cardiovascular diseases, such as hypertension, cardiac hypertrophy, heart failure and ischemia-reperfusion injury, by promoting ROS generation (Cave et al., 2006). Therefore, it was speculated that jaranol had a protective effect on cardiovascular disease. Although Jing-Ting Huang’s study investigated the pharmacokinetics of jaranol in rats, the pharmacodynamics and toxicity of jaranol remain unclear (Huang et al., 2017). In the current study, the aim was to evaluate the acute and sub-acute toxicity of jaranol in mice, which will provide a critical basis for advanced preclinical studies on the pharmacodynamics and mechanism of jaranol.

## 2 Materials and Methods

### 2.1 Chemicals and Reagents

Jaranol (NO.3301-49-3) was obtained from Alfabiotech (Chengdu, China). Jaranol is a yellow powder with a final purity of at least 98% and stored at 4°C. The chromatograms, LC/MS spectrum and nuclear magnetic resonance spectrum of jaranol were shown in Supplementary Figures S1–S3. Before gavage, jaranol was dissolved in 0.5% carboxymethyl cellulose sodium (CMC-Na) to prepare a suspension.

### 2.2 Experimental Animals

Five-week-old Kunming mice were obtained from the Laboratory Animal Centre of Inner Mongolia University (License ID: SCXK 2016-0001, Hohhot, China). The animals were housed under 12 h light/dark cycles at 23–25°C in a 40%–70% relative humidity environment. All animals had free access to water and food during the experimental periods. The animals were acclimatized to the laboratory conditions for a week prior to experiments. The animal experiments were performed in accordance with the Principles of Laboratory Animal Care and approved by the ethical committee of experimental animal care at Inner Mongolia Medical University.

### 2.3 Acute Toxicity Study

It was done as per Organization for Economic Co-operation and Development (OECD) guidelines 423 for acute toxicity studies. Twelve mice were divided into 2 groups of 6 mice each (three males and three females), and the mice were fasted for 24 h prior to experiments. One group was given a single dose of 2,000 mg/kg body weight (BW) jaranol by gavage. Six other mice (three males and three females) received 0.5% CMC-Na with 1 ml/100 g BW as a control. The animals were observed for any clinical signs at least once during the first 30 min, periodically during the first 24 h, with special attention given during the first 4 h, and daily thereafter, for a total of 14 days. Main clinical signs included toxicity, mortality, behavioral pattern, and physical appearance, as well as other adverse effects, for example lethargy, diarrhea, tremors, salivation, etc.

### 2.4 Sub-Acute Toxicity Study

Sub-acute toxicity study of jaranol in mice was performed as indicated by a slightly modified OECD guidelines 407. Twenty-four mice were divided into 4 groups (three males and three females) to evaluate sub-acute toxicity of Jaranol (Ishtiaq et al., 2017). The experimental groups were given jaranol at graded dose of 50, 100 or 200 mg/kg BW by gavage for 28 consecutive days. The doses were selected according to Schedule Y (The Drugs and Cosmetics Rules, 1945; India) on the basis of ED_50_ value (Rayees et al., 2013). The control treatment was 0.5% CMC-Na with 1 ml/100 g BW as a control. The mice were weighed weekly and observed for toxic reactions and mortality. Food intake and water consumption were recorded weekly. At the end of the experiments, the mice were anaesthetized with 2% tribromoethanol (T484020, Sigma–Aldrich, St. Louis, MO, United States, 3 ml/20 g BW). Previous study showed that white blood cells parameters, total red blood cells and platelets, hematocrit, and hemoglobin concentration were equivalent for cardiac and retro-orbital bleeding. Therefore, retro-orbital bleeding was selected in this study (Hoggatt et al., 2016). Blood was collected retro-orbitally into tubes containing EDTA (ethylenediaminetetraacetic acid) for hematological study and without EDTA for biological study, and then mice were sacrificed. Mouse major organs, including the heart, kidney, spleen, lung and liver, were harvested to calculate organ coefficients. The organ coefficients of each animal were calculated by the following formula: organ coefficient (g/100 g) = [organ wet weight (g)/body weight (g)] × 100.

### 2.5 Hematological Study

Blood samples were collected into tubes containing EDTA and were sent to Lab -Test Biotechnology Ltd., Co (Beijing, China) for analyzing hematological parameters using an automatic veterinary blood cell analyzer (BC-5000Vet, CBIO Science and Technology, Beijing, China). Hematological parameters included WBC (white blood cells), NETU (neutrophils), LYMPH (lymphocytes), MONO (monocytes), EO (eosinophil), BASO (basophils), NEU (neutrophils), RBC (red blood cell), HGB (hemoglobin), HCT (hematocrit), MCV (mean corpuscular volume), MCH (melanin-concentrating hormone), MCHC (mean corpuscular hemoglobin concentration), RDW-CV (red cell distribution width-coefficient of variation), RDW-SD (red cell distribution width-standard deviation), PLT (platelet), MPV (mean platelet volume), PDW (platelet distribution width), and PCT (procalcitonin).

### 2.6 Biochemical Parameters

Blood samples were also collected into tubes without EDTA. The samples were then centrifuged at 5,000 RPM for 10 min and the serum was then removed and stored at −80 °C until analysis. Serum samples were analyzed by an automatic biochemical analyzer (Ichem-340, ICUBIO, Shenzhen, China) to obtain biochemical parameters, including LDL-C (low-density lipoprotein cholesterol), HDL-C (high-density lipoprotein cholesterol), CHO (cholesterol), ALT (alanine aminotransferase), AST (aspartate aminotransferase), T-Bil (total bilirubin), and Cre (Creatinine).

### 2.7 Histopathology

The histological study of the heart, kidney, spleen, lung and liver were performed as described previously (Leong et al., 2018). Briefly, tissues were fixed in 10% paraformaldehyde and then embedded in paraffin wax after the samples were dehydrated in graded alcohol. Samples were cut transversely into 5 μm sections. Serial sections were stained with a hematoxylin-eosin (HE) staining kit (Yuanye Bio-Technology, R20570, Shanghai, China) to evaluate tissue damage. At least two pathologists (L-TL and D-Y) scored them simultaneously under microscope and awarded a graded expert staining score from 1 to 5 points based on staining quality. The scoring criteria as following: 5, excellent staining; 4, pass with minor remark; 3, deficiency without affecting clinical output; 2, incorrect staining with clinical output affected; and 1, failed staining, impossible to interpret (Keppens et al., 2020).

### 2.8 Statistical Analyses

The statistical software IBM SPSS 20 (SPSS Inc., Chicago, IL) was used to perform the statistical analysis. All data were presented as the mean ± SD. Statistical differences were calculated with the 2-tailed Student *t* test when comparing 2 conditions, and ANOVA was used when comparing >2 conditions. For parametric data with equal variance, one-way ANOVA with Tukey *post-hoc* test was used. For parametric data with unequal variance, one-way ANOVA with the Games-Howell *post-hoc* test was used (Liu et al., 2020b). Data were considered statistically significant at *p* < 0.05.

## 3 Results

### 3.1 Acute Toxicity Study

During the acute toxicity test, there were no signs of toxicity observed in the autonomic and central nervous systems, skin (fur), water consumption and food intake after administration of jaranol at a single dose of 2,000 mg/kg BW for 14 days (Supplementary Figure S4). No mortality was recorded during this time. No alterations were found in organ coefficient, hematological parameters, kidney function and lipid profile. Adverse effects of jaranol on mouse liver were observed in acute toxicity, which illustrated by a significant decrease in serum alanine aminotransferase and aspartate aminotransferase level of jaranol-treated mice compared to control. However, we observed no significant change in the macroscopic and microscopic structure of the liver between jaranol-treated mice and control. Those results showed that the oral lethal dose 50 (LD_50_) of jaranol was higher than 2,000 mg/kg BW, regardless of sex (Supplementary Tables S1–S4).

### 3.2 Sub-Acute Toxicity Study

#### 3.2.1 Effects of Jaranol on Body Weight, Food and Water Intake

**FIGURE 1:**
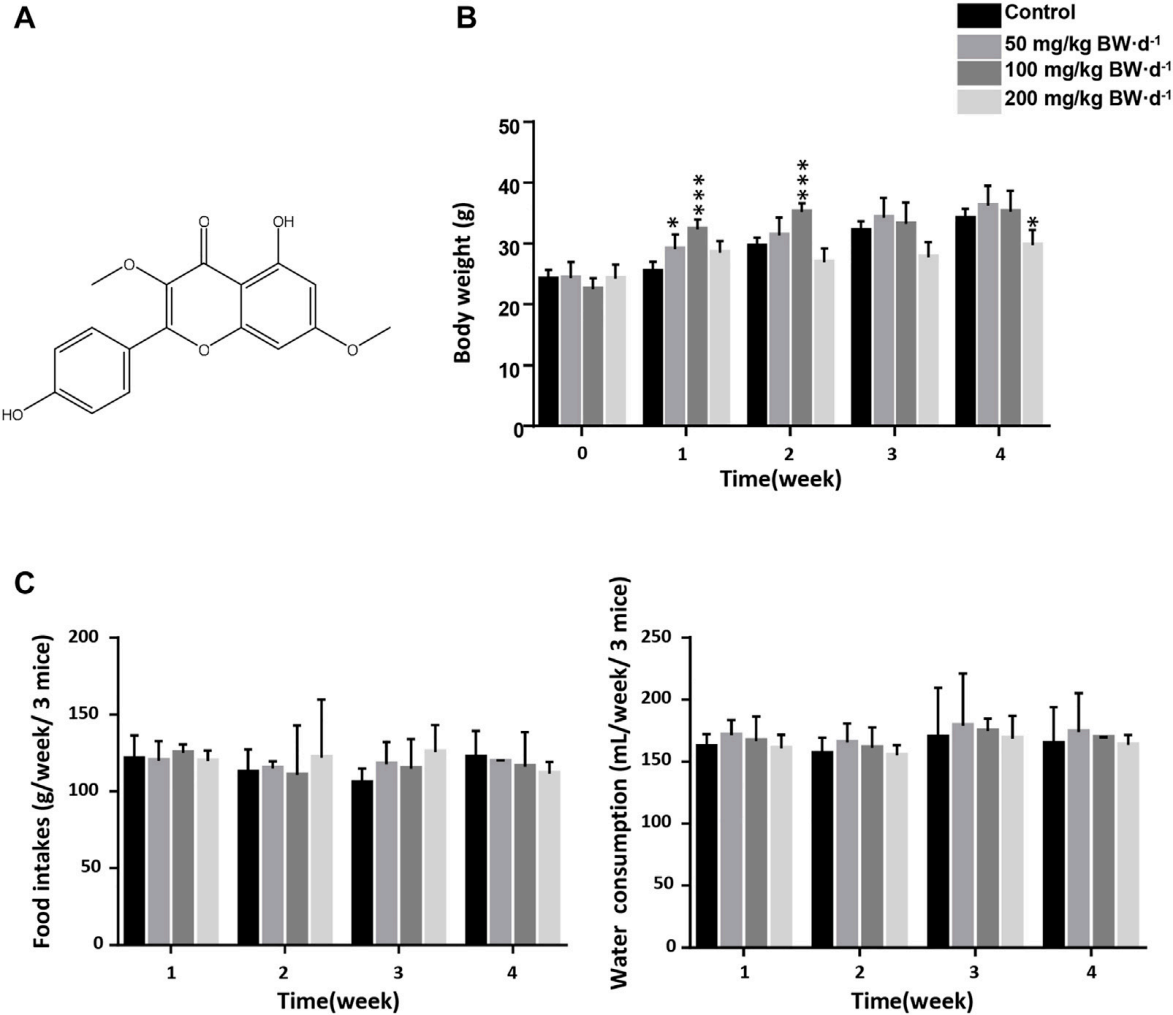
Effect of daily oral administration of jaranol on body weight, food intake and water consumption in mice. **(A)** Chemical structure of jaranol. **(B)** Mouse body weights of different groups (n = 6). One-way One-way ANOVA followed by Tukey *post-hoc* test, compared with Control, ^*^ *p* < 0.05, ^***^ *p* < 0.001. **(C)** Food intake and water consumption in mice, n = 2 cage, 3 mice/cage.

The chemical structure of jaranol is shown in Figure 1A. To evaluate the sub-acute toxicity of jaranol in mice, mice were administered 50, 100 or 200 mg/kg BW jaranol per day by gavage for 28 days. No mortality or toxic signs were observed during the study of sub-acute toxicity. A gradual significant increase in body weight was found in the control group, and body weight gain was significantly inhibited in 200 mg/kg BW jaranol-treated mice (Figure 1B). During the experiment, food intake and water consumption were recorded weekly. The results showed no significant change in the food intake and water consumption of the mice in all groups (Figure 1C).

### 3.3 Effects of Jaranol on Organ Coefficients

After the mice were treated by jaranol for 28 days, mouse major organs, including the heart, kidney, spleen, lung and liver, were harvested and weighted, and then the organ coefficient was calculated. Organ weight data was shown in Supplementary Table S5. A significant decrease in the ratio of spleen weight to body weight was found in all jaranol-treated mice, and 200 mg/kg BW·d^−1^ jaranol-treated mice had a significant decrease in the ratio of liver weight to body weight compared to control. No statistically significant difference in the ratio of heart, kidney and lung weight to body weight was found between the control and jaranol-treated mice (Table 1). Female mice administered jaranol at a dosage of 200 mg/kg BW·d^−1^ by gavage for 28 days had a significant increase in the ratio of lung weight to body weight compared to control. Except for lung, the effect of jaranol on organ coefficient were no any differences between the sexes (Supplementary Table S6).

**TABLE 1:** Effect of daily oral administration of jaranol on organ coefficient in mice.

Parameters	Control	50 mg/kg BW·d^−1^	100 mg/kg BW·d^−1^	200 mg/kg BW·d^−1^
Heart (g/100 g BW)	0.54 ± 0.099	0.436 ± 0.06	0.467 ± 0.079	0.444 ± 0.08
Liver (g/100 g BW)	5.476 ± 1.091	4.639 ± 0.377	4.777 ± 0.664	3.821 ± 0.504^**^
Spleen (g/100 g BW)	0.361 ± 0.089	0.263 ± 0.025^*^	0.248 ± 0.051^*^	0.229 ± 0.04^**^
Lung (g/100 g BW)	0.592 ± 0.072	0.524 ± 0.078	0.528 ± 0.074	0.501 ± 0.075
Kidney (g/100 g BW)	0.537 ± 0.09	0.492 ± 0.054	0.465 ± 0.064	0.692 ± 0.65

All data are reported as the mean ± SD, for n = 6 per group. One-way ANOVA, followed by Tukey *post-hoc* test, compared with Control, ^*^ *p* < 0.05, ^**^ *p* < 0.01.

### 3.4 Effects of Jaranol on Hematological Parameters

To further evaluate the sub-acute toxicity of jaranol, mouse hematological parameters were detected by an automatic veterinary blood cell analyzer after treatment for 28 days. Our results showed that 50 mg/kg BW·d^−1^ jaranol treatment by gavage had no significant effect on hematological parameters compared with the control. Mice administered by jaranol at a dosage of 100 mg/kg BW·d^−1^ by gavage for 28 days had a significant increase in RBCs, while MCV and MCH levels were significantly decreased compared to the control. Meanwhile, a significant increase in RBCs was also found in 200 mg/kg BW·d^−1^ jaranol-treated mice compared to control mice (Table 2). In conclusion, we considered that 100 and 200 mg/kg BW·d^−1^ jaranol treatment by gavage for 28 days had a significant effect on RBCs in mice. For female mice, high dose (100 and 200 mg/kg BW·d^−1^) jaranol administered by gavage for 28 days had a significant effect on neutrophil and lymphocyte count, the effects of jaranol on others hematological parameters were not significantly differential between the sexes (Supplementary Table S7).

**TABLE 2:** Effect of daily oral administration of jaranol on hematological parameters in mice.

Parameters	Control	50 mg/kg BW·d^−1^	100 mg/kg BW·d^−1^	200 mg/kg BW·d^−1^
WBC (10^9/L)	6.27 ± 1.44	4.29 ± 1.63	4.87 ± 1.69	5.52 ± 1.27
NETU (10^9/L)	1.59 ± 0.62	1.16 ± 0.84	1.2 ± 0.67	1.51 ± 0.43
LYMPH (10^9/L)	3.88 ± 0.96	2.73 ± 0.97	3.24 ± 1.06	3.56 ± 0.96
MONO (10^9/L)	0.29 ± 0.11	0.23 ± 0.14	0.23 ± 0.07	0.24 ± 0.09
EO (10^9/L)	0.47 ± 0.48	0.14 ± 0.11	0.19 ± 0.09	0.18 ± 0.13
BASO(10^9/L)	0.04 ± 0.03	0.03 ± 0.02	0.04 ± 0.02	0.03 ± 0.02
NETU (%)	26.43 ± 10.91	25.05 ± 14.73	22.72 ± 9.89	27.4 ± 6.26
LYMPH (%)	61.77 ± 5.26	65.25 ± 14.59	68.22 ± 10.51	64 ± 5.97
MONO (%)	4.47 ± 0.92	5.43 ± 2.77	4.05 ± 1.84	4.42 ± 1.2
EO (%)	6.63 ± 6.16	3.43 ± 2.72	3.97 ± 1.28	3.53 ± 2.75
BASO (%)	0.70 ± 0.28	0.83 ± 0.46	1.05 ± 0.6	0.65 ± 0.37
RBC(10^12/L)	8.61 ± 0.47	8.96 ± 1.02	9.69 ± 0.99^*^	9.31 ± 0.52^*^
HGB (g/L)	150.33 ± 6.56	154.5 ± 14.49	163.67 ± 18.37	158.33 ± 8.8
HCT (%)	43.83 ± 2.75	44.2 ± 5.26	46.28 ± 4.82	44.63 ± 2.43
MCV (FL)	50.95 ± 2.98	49.37 ± 1.77	47.82 ± 2.06^*^	47.9 7 ± 0.92
MCH(pg)	17.45 ± 0.33	17.3 ± 0.42	16.85 ± 0.69^*^	17.02 ± 0.34
MCHC (g/L)	344 ± 18.42	350.5 ± 14.49	353 ± 8.74	355.33 ± 8.33
RDW-CV(%)	15.78 ± 1.89	18.87 ± 4.46	17.85 ± 2.91	16.05 ± 2.35
RDW-SD (fl)	35.8 ± 6.16	41.5 ± 10.84	38.22 ± 7.83	34.4 ± 5.06
PLT (10^9/L)	393.5 ± 137.82	312 ± 126.22	403.5 ± 169.44	276.33 ± 85.89
MPV(fl)	5.62 ± 0.31	5.88 ± 0.38	5.7 ± 0.53	5.52 ± 0.33
PDW (fl)	16.42 ± 0.37	16.63 ± 0.25	16.42 ± 0.35	16.27 ± 0.29
PCT (%)	0.22 ± 0.08	0.18 ± 0.07	0.23 ± 0.09	0.15 ± 0.05

All data are reported as the mean ± SD. for n = 6 per group. One-way ANOVA followed by Tukey *post-hoc* test, compared with Control, ^*^ *p* < 0.05. WBC, white blood cells; NETU, neutrophils; LYMPH, lymphocytes; MONO, monocytes; EO, eosinophil; BASO, basophils; NEU, neutrophils; RBC, red blood cell; HGB, hemoglobin; HCT, hematocrit; MCV, mean corpuscular volume; MCH, melanin-concentrating hormone; MCHC, mean corpuscular hemoglobin concentration; RDW-CV, red cell distribution width-coefficient of variation; RDW-SD, red cell distribution width-standard deviation; PLT, platelet; MPV, mean platelet volume; PDW, platelet distribution width; PCT, procalcitonin.

### 3.5 Effects of Jaranol on Liver and Kidney Function

Serum ALT, AST and T-Bil levels were measured by an automatic biochemical analyzer to evaluate mouse liver function. The results showed a significant decrease in serum ALT levels in mice that received jaranol treatment at dose of 200 mg/kg BW·d^−1^ for 28 days compared with control, especially for male mice. Serum AST and T-Bil levels had not significantly difference between control and jaranol-treated mice (Table 3 and Supplementary Table S8). For kidney function, mice administered 200 mg/kg BW·d^−1^ jaranol treatment for 28 days had a significant increase in serum Cre levels compared with control (Table 3). The effects of jaranol on kidney function were not significantly difference between the sexes (Supplementary Table S8). These results indicate that chronic (28 days) oral administration of jaranol (200 mg/kg BW·d^−1^) has an effect on liver and kidney function.

**TABLE 3:** Effect of daily oral administration of jaranol on liver and kidney function in mice.

Parameters	Control	50 mg/kg BW·d^−1^	100 mg/kg BW·d^−1^	200 mg/kg BW·d^−1^
ALT (U/L)	27.33 ± 5.09	32.57 ± 17.74	21.34 ± 4.31	18.52 ± 3.1^**^
AST (U/L)	85.23 ± 12.99	158.25 ± 91.46	79.14 ± 16.76	72.34 ± 14.48
T-Bil (μmol/L)	13.1 ± 2.23	11.43 ± 3.38	13.44 ± 2.91	15.02 ± 3.09
Cre(μmol/L)	18.83 ± 2.89	19.78 ± 1.32	18.89 ± 3.54	26.34 ± 3.54^**^

All data are reported as the mean ± SD, for n = 6 per group. One-way ANOVA, followed by Tukey *post-hoc* test, compared with Control, ^**^ *p* < 0.01. ALT, alanine aminotransferase; AST, aspartate aminotransferase; T-Bil, total bilirubin; Cre, Creatinine.

### 3.6 Effects of Jaranol on the Lipid Profile

After jaranol treatment for 28 days, serum lipid profile was detected by an automatic biochemical analyzer. The effects of jaranol on the lipid profile are presented in Table 4. The results showed that no significant change in serum HDL-C, LDL-C or CHO levels was observed between control and jaranol-treated mice after treatment for 28 days. Moreover, the effects of jaranol on lipid profile had not significantly difference between the sexes (Supplementary Table S9).

**TABLE 4:** Effect of daily oral administration of jaranol on lipid profile in mice.

Parameters	Control	50 mg/kg BW·d^−1^	100 mg/kg BW·d^−1^	200 mg/kg BW·d^−1^
LDL-C (mmol/L)	1.51 ± 0.29	1.77 ± 0.49	1.26 ± 0.11	1.4 ± 0.21
HDL-C (mmol/L)	2.02 ± 0.32	2.3 ± 0.74	2.1 ± 0.34	2.45 ± 0.75
CHO(mmol/L)	2.6 ± 0.38	2.99 ± 0.89	2.35 ± 0.31	2.62 ± 0.55

All data are reported as the mean ± SD, for n = 6 per group. LDL-C, low-density lipoprotein cholesterol; HDL-C, high-density lipoprotein cholesterol; CHO, cholesterol; ANOVA, one way followed by Tukey *post-hoc* test.

### 3.7 Effects of Jaranol on Histopathology

**FIGURE 2:**
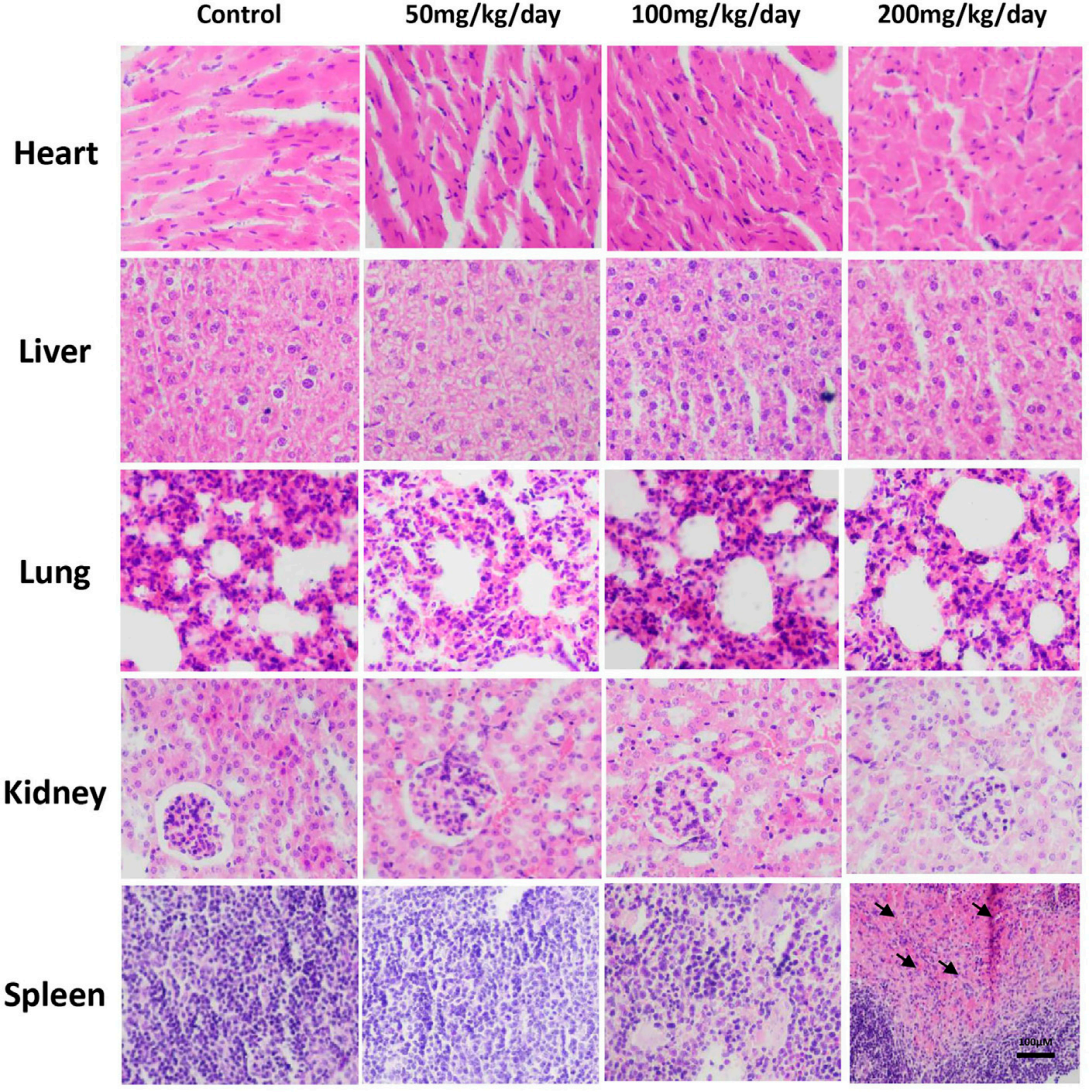
Histopathological study of mouse major organs following oral administration of jaranol for 28 days. Scale bar = 100 μM (black arrow: bleeding).

At the end of treatment, major organs, including the heart, liver, spleen, lung and kidney, were subjected to histopathological evaluation to provide evidence supporting the findings of the biochemical assessments. Graded expert staining score was obtained by two pathologists to evaluate staining quality, which was shown in Supplementary Table S10. According to the results, no significant change was found in the histopathological examination of the lung, heart, liver, and kidneys of the jaranol-treated groups compared to control (Figure 2). The macroscopic evaluations showed that no lesions, inflammation or pathological changes were determined in the lung, spleen, heart, liver and kidneys due to the jaranol administration. Moreover, the histopathological evaluation of kidneys, spleen, liver, lung and heart showed no necrosis and were consistent to control. However, histological study revealed the spleen bleeding was identified in jaranol-treated mice at dose of 200 mg/kg BW·d^−1^ by gavage for 28 days (Figure 2).

## 4 Discussion

Previous studies indicated that flavonoids had a significant effect on increasing RBCs in normal and diabetic rat, which was consistent with our results (Mahmoud 2013; Gao et al., 2017). Indeed, Ken Y Z Zheng’s study showed that flavonoids from *Radix Astragali* could promote the expression of erythropoietin *in vitro*, and erythropoietin is an important hormone that could stimulate stem cells in the bone marrow to produce red blood cells (Ohlsson and Aher 2006; Zheng et al., 2011). In addition, the effect of jaranol on RBCs may be attributed to its anti-oxidant activity. It was reported that flavonoids could decrease lipid peroxidation levels, which leads to hemolysis of erythrocytes (Mahmoud et al., 2012). In our study, a significant increase in RBC counts was found in jaranol-treated mice; however, MCH and MCHC levels were not significantly differential compared to the control mice. These results illustrate that the effect of jaranol on RBC counts was related to its anti-oxidant activity. The detailed mechanism needs further research.

Our results showed that 200 mg/kg BW·d^−1^ jaranol-treated mice had a significantly decreased serum ALT level. However, it was not clear whether decreased serm ALT levels could reflect the protective and adverse effects of jaranol on liver function under normal pathological conditions. Similar results were also found in other studies (Gao et al., 2017; Erdenetsogt et al., 2020). Previous studies showed that flavonoids were mainly metabolized by phase I and phase II metabolism in the liver and gastrointestinal tract. Although flavonoids have been reported to have a protective effect on multiple liver injuries, intake of flavonoids at high dosages causes a liver metabolic burden (Liu et al., 2020a). In addition, flavonoids could regulate the activity of hepatic cytochrome CYP450, and cytochrome CYP450 signaling was involved in the pathological process of liver injury (Ekstrand et al., 2015; Li et al., 2021). At present, there are no reports detailing kidney injury induced by flavonoids. Some studies have shown that kidney injury is improved by the administration of flavonoids from different sources (Park et al., 2020). However, flavonoids could reduce the elimination of nephron cardiovascular toxins by impairing renal function (Vargas et al., 2018). In this study, a significant change in serum Cre levels was found in 200 mg/kg BW·d^−1^ jaranol-treated mice, which may be attributed to jaranol-induced alterations to the kidney elimination process.

## 5 Conclusion

A study of acute toxicity indicated that a single administration of jaranol by the oral route did not cause any lethality or any changes in general behavior in mice. However, regarding sub-acute toxicity, the results showed that jaranol treatment at a dosage of 200 mg/kg BW·d^−1^ by gavage had a significant effect on RBCs and liver and kidney function. This studies are the first studies of this nature with jaranol, and provide important toxicological information to be used for further preclinical studies on the pharmacodynamics and mechanism of action.

## Data Availability

The original contributions presented in the study are included in the article/Supplementary Materials, further inquiries can be directed to the corresponding authors.
