# Supplementary material for: Evaluation of the Acute and Sub-Acute Oral Toxicity of Jaranol in Kunming Mice
Source: Front Pharmacol. 2022 Jun 30;13:903232. doi: 10.3389/fphar.2022.903232 (PMC9280858; doi:10.3389/fphar.2022.903232)
Supplement: Supplementary file 1 [file DataSheet1.docx]

*
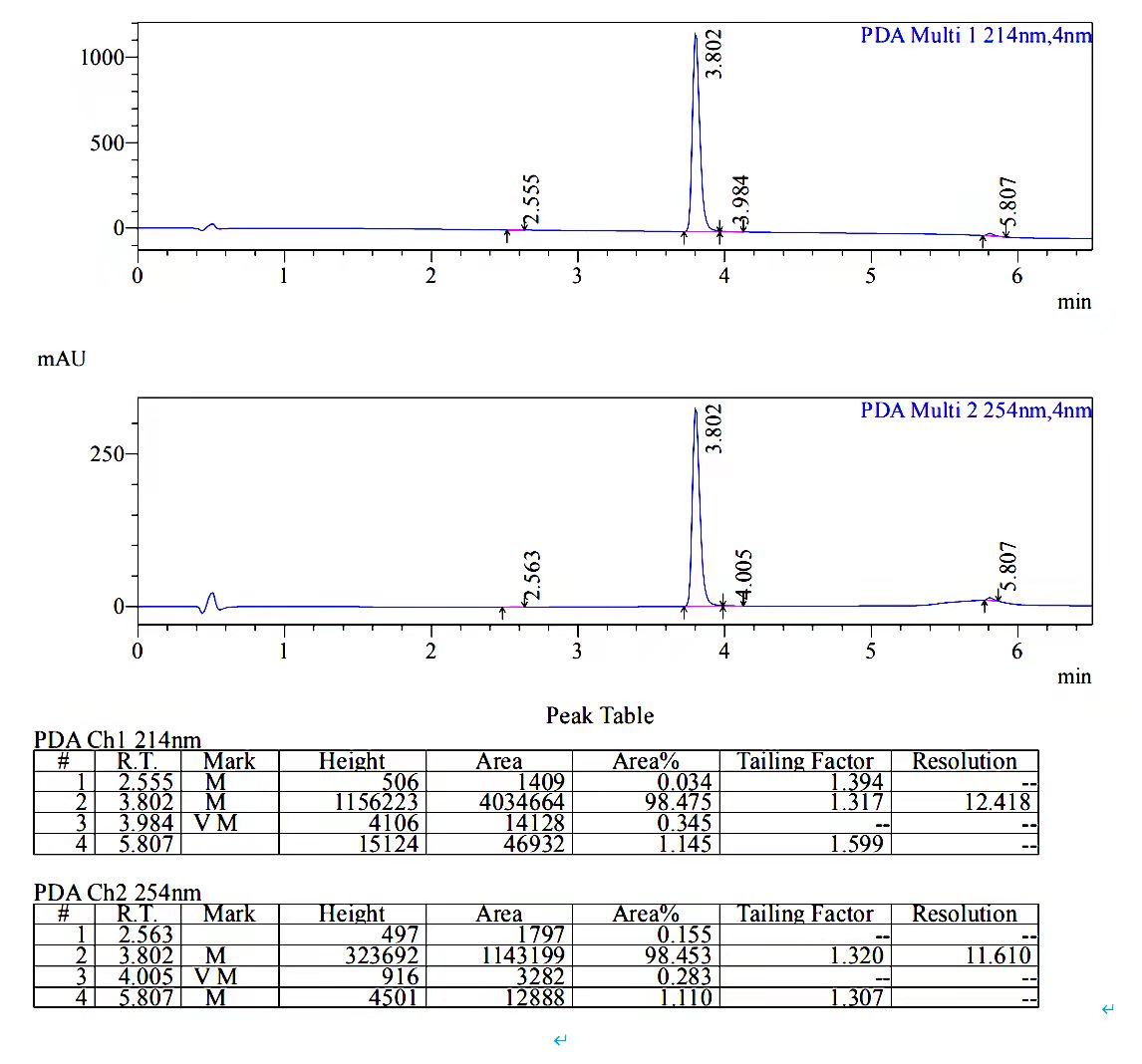
*

Figure S1 Chromatograms of Jaranol

*
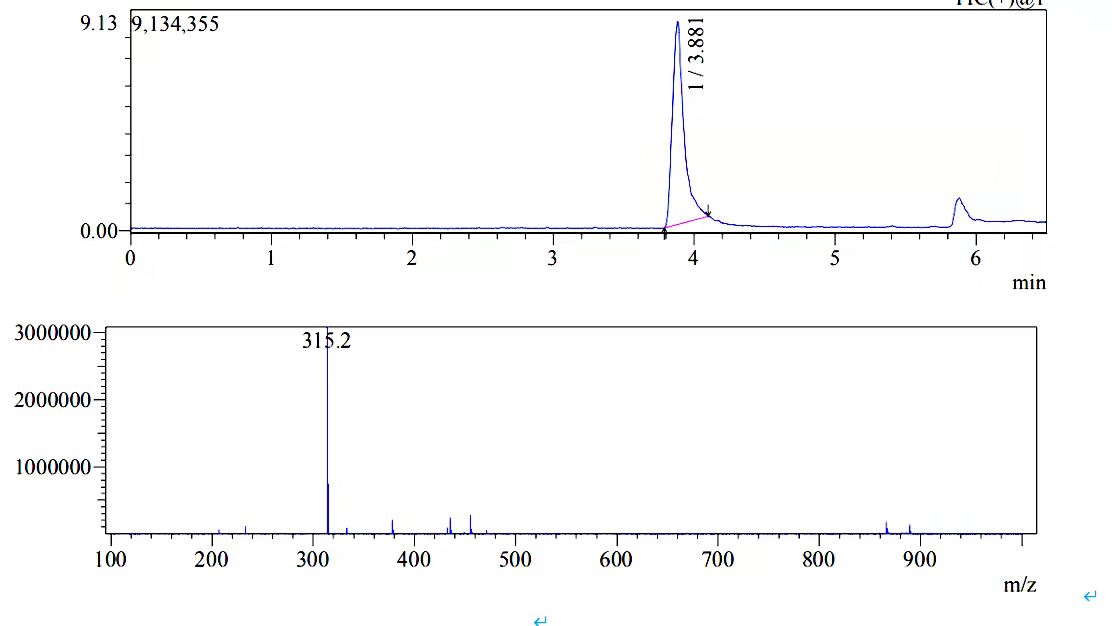
*

Figure S2 LC/MS spectrum of Jaranol


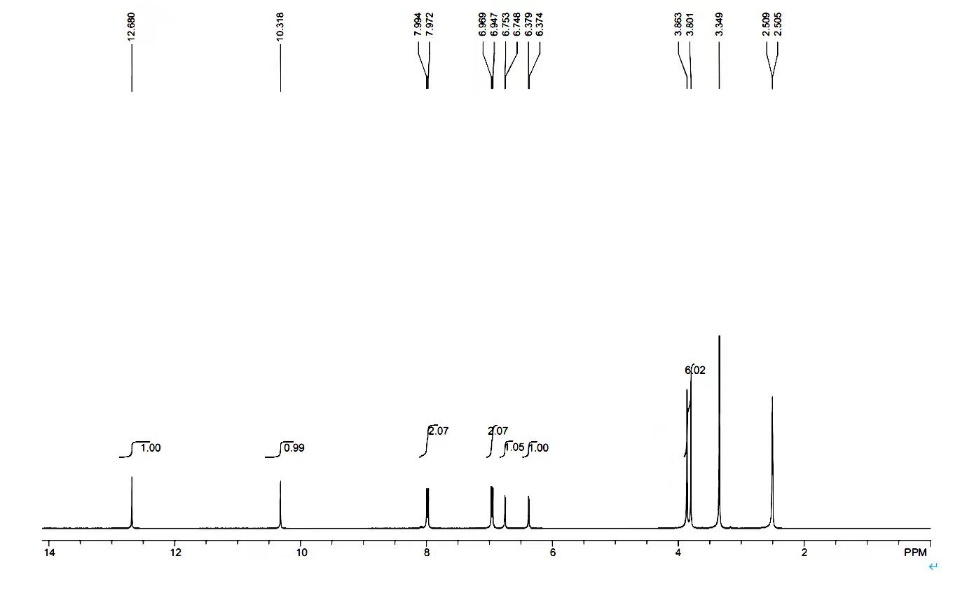


Figure S3 Nuclear magnetic resonance (NMR) spectrum of Jaranol


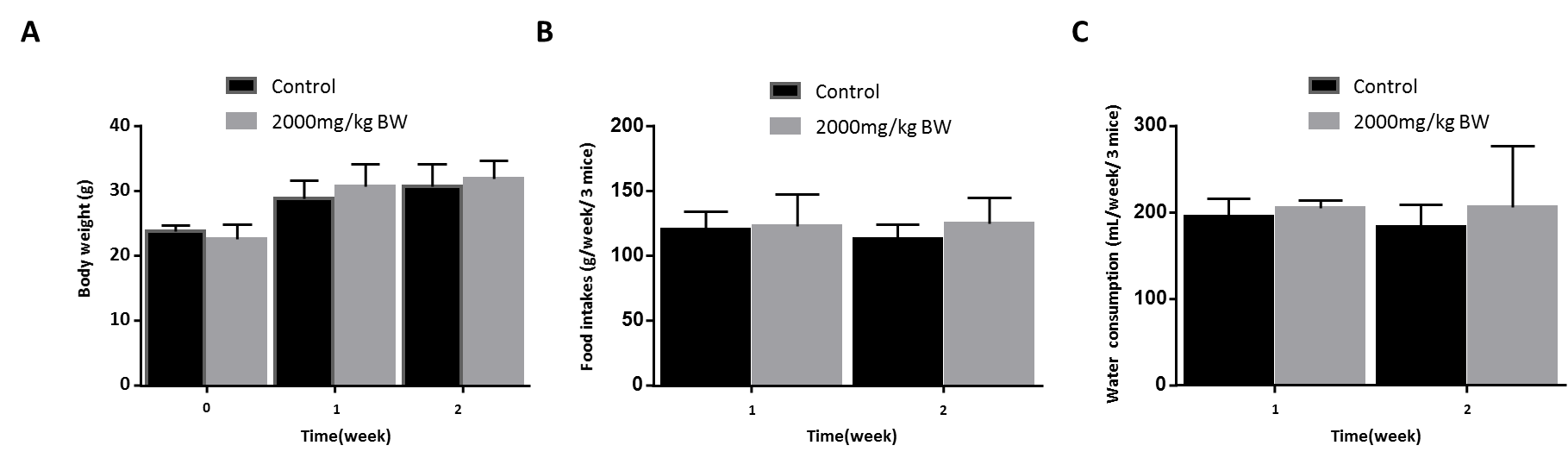


Figure S4 Effect of a single dose of 2000 mg/kg BW jaranol on body weight (A), food intakes (B) and water consumption

All data are reported as the mean ± SD. for n=6 per group. Student’s *t* test, compared with Control.

Table S1 Effect of a single dose of 2000 mg/kg BW jaranol on organ coefficient in mice

| Parameters | Control | 2000 mg/kg BW |
| --- | --- | --- |
| Heart (g/100g BW) | 0.53±0.04 | 0.574±0.067 |
| Liver (g/100g BW) | 4.26±0.55 | 4.558±0.497 |
| Spleen(g/100g BW) | 0.30±0.05 | 0.296±0.03 |
| Lung (g/100g BW) | 0.56±0.04 | 0.536±0.037 |
| Kidney(g/100g BW) | 0.41±0.06 | 0.489±0.078 |

All data are reported as the mean ± SD. for n=6 per group. Student’s *t* test, compared with Control.

Table S2 Effect of a single dose of 2000 mg/kg BW jaranol on hematological parameters in mice

| Parameters | control | 2000 mg/kg BW |
| --- | --- | --- |
| WBC(10^9/L) | 3.13±0.9 | 4.86±2.89 |
| NETU(10^9/L) | 0.45±0.32 | 1±0.5 |
| LYMPH(10^9/L) | 2.12±0.45 | 3.44±2.22 |
| MONO(10^9/L) | 0.14±0.05 | 0.17±0.09 |
| EO(10^9/L) | 0.31±0.32 | 0.2±0.14 |
| BASO(10^9/L) | 0.05±0.02 | 0.04±0.04 |
| NETU (%) | 20.1±1.77 | 20.66±8.82 |
| LYMPH(%) | 69.15±12.61 | 70.2±7.92 |
| MONO(%) | 4.75±2.11 | 3.68±1.3 |
| EO(%) | 4.33±2.44 | 4.4±1.72 |
| BASO(%) | 0.89±0.78 | 1.06±0.54 |
| RBC(10^12/L) | 7.28±0.53 | 8.58±1.46 |
| HGB(g/L) | 146.16±7.22 | 149.2±19.22 |
| HCT(%) | 40.13±3.64 | 40.98±5.82 |
| MCV(FL) | 49.43±4.2 | 48±2.54 |
| MCH(pg) | 18.5±2.8 | 17.48±1.04 |
| MCHC(g/L) | 365.66±22.99 | 364.4±7.02 |
| RDW-CV(%) | 17.28±3.27 | 16.98±3.57 |
| RDW-SD(fl) | 35.01±6.06 | 35.96±8.89 |
| PLT(10^9/L) | 340.5±174.63 | 301.4±77.64 |
| MPV(fl) | 5.28±2.17 | 5.74±0.48 |
| PDW(fl) | 16.43±0.97 | 16.42±0.3 |
| PCT(%) | 0.35±0.27 | 0.17±0.03 |

All data are reported as the mean ± SD. for n=6 per group. Student’s *t* test, compared with Control. WBC, white blood cells; NETU, neutrophils; LYMPH, lymphocytes; MONO, monocytes; EO, eosinophil; BASO, basophils; NEU, neutrophils; RBC, red blood cell; HGB, hemoglobin; HCT, hematocrit; MCV, mean corpuscular volume; MCH, melanin-concentrating hormone; MCHC, mean corpuscular hemoglobin concentration; RDW-CV, red cell distribution width- coefficient of variation; RDW-SD, red cell distribution width- standard deviation; PLT, platelet; MPV, mean platelet volume; PDW, platelet distribution width; PCT, procalcitonin.

Table S3 Effect of a single dose of 2000 mg/kg BW jaranol on liver and kidney function in mice

| Parameters | control | 2000 mg/kg BW |
| --- | --- | --- |
| ALT(U/L) | 39.91±5.65 | 19.67±5.68* |
| AST(U/L) | 71.15±12.5 | 61.54±10.23* |
| T-Bil(μmol/L) | 13.43±2.61 | 16.46±1.65 |
| Cre(μmol/L) | 21.24±15.61 | 19.81±1.73 |

All data are reported as the mean ± SD. for n=6 per group. Student’s *t* test, compared with Control, ^*^*P*<0.05. ALT, alanine aminotransferase; AST, aspartate aminotransferase; T-Bil, total bilirubin; Cre, Creatinine.

Table S4 Effect of a single dose of 2000 mg/kg BW jaranol on lipid profile in mice

| Parameters | control | 2000 mg/kg BW |
| --- | --- | --- |
| LDL-C(mmol/L) | 1.57±0.24 | 1.24±0.23 |
| HDL-C(mmol/L) | 2.5±0.84 | 2.29±0.74 |
| CHO(mmol/L) | 2.81±0.38 | 2.39±0.57 |

All data are reported as the mean ± SD. for n=6 per group. LDL-C, low-density lipoprotein cholesterol; HDL-C, high-density lipoprotein cholesterol; CHO, cholesterol. Student’s *t* test, compared with Control.

Table S5 Organ weight data of different groups

|  | control | 50mg/kg/day | 100mg/kg/day | 200mg/kg/day |
| --- | --- | --- | --- | --- |
| heart | 0.17±0.029 | 0.12±0.012 | 0.16±0.039 | 0.15±0.029 |
| liver | 1.75±0.294 | 1.05±0.124 | 1.6±0.332 | 1.58±0.121 |
| spleen | 0.12±0.028 | 0.06±0.015 | 0.08±0.017 | 0.09±0.01 |
| lung | 0.19±0.015 | 0.15±0.026 | 0.17±0.017 | 0.17±0.014 |
| kidney(left) | 0.17±0.026 | 0.14±0.02 | 0.15±0.025 | 0.24±0.222 |

All data are reported as the mean ± SD. for n=6 per group.

Table S6 Effect of daily oral administration of jaranol on organ coefficient in mice

| Parameters | Male (n=3) | | | | Female (n=3) | | | |
| --- | --- | --- | --- | --- | --- | --- | --- | --- |
|  | Control | 50mg/kg/day | 100mg/kg/day | 200mg/kg/day | Control | 50mg/kg/day | 100mg/kg/day | 200mg/kg/day |
| Heart (g/100g body weigh) | 0.509±0.125 | 0.392±0.021 | 0.409±0.037 | 0.426±0.036 | 0.571±0.076 | 0.479±0.053 | 0.525±0.065 | 0.461±0.117 |
| Liver (g/100g body weigh) | 5.653±1.317 | 3.785±0.499 | 5.176±0.42 | 4.713±0.033 | 5.299±1.07 | 3.858±0.619 | 4.378±0.669 | 4.564±0.581 |
| Spleen(g/100g body weigh) | 0.347±0.104 | 0.222±0.055 | 0.26±0.066 | 0.259±0.028 | 0.374±0.091 | 0.236±0.028 | 0.235±0.042 | 0.266±0.028 |
| Lung (g/100g body weigh) | 0.566±0.037 | 0.51±0.033 | 0.545±0.096 | 0.561±0.049 | 0.619±0.098 | 0.537±0.117 | 0.511±0.059 | 0.44±0.029^*^ |
| Kidney(g/100g body weigh) | 0.534±0.086 | 0.474±0.03 | 0.435±0.084 | 0.96±0.916 | 0.54±0.113 | 0.511±0.074 | 0.494±0.023 | 0.423±0.019 |

All data are reported as the mean ± SD. for n=6 per group. One-way ANOVA followed by Games-Howell *post hoc* test, compared with Control, ^*^*P*<0.05.

Table S7 Effect of daily oral administration of jaranol on hematological parameters in mice

| Parameters | Male (n=3) | | | | Female (n=3) | | | |
| --- | --- | --- | --- | --- | --- | --- | --- | --- |
|  | control | 50mg/kg/day | 100mg/kg/day | 200mg/kg/day | control | 50mg/kg/day | 100mg/kg/day | 200mg/kg/day |
| WBC(10^9/L) | 6.39±1.76 | 3.18±0.6* | 5.37±1.15 | 5.13±1.62 | 6.15±1.42 | 5.4±1.61 | 4.36±2.26 | 5.91±0.98 |
| NETU(10^9/L) | 1.05±0.06 | 0.45±0.21** | 1.57±0.24 | 1.51±0.57 | 2.14±0.28 | 1.87±0.43 | 0.83±0.79 | 1.5±0.37 |
| LYMPH(10^9/L) | 4.22±1.09 | 2.4±0.46 | 3.32±0.98 | 3.12±1.22 | 3.53±0.87 | 3.05±1.35 | 3.15±1.36 | 4±0.49 |
| MONO(10^9/L) | 0.33±0.14 | 0.14±0.05 | 0.27±0.07 | 0.24±0.06 | 0.25±0.1 | 0.33±0.14 | 0.18±0.01 | 0.24±0.12 |
| EO(10^9/L) | 0.74±0.56 | 0.16±0.12 | 0.18±0.1 | 0.23±0.18 | 0.19±0.17 | 0.12±0.12 | 0.19±0.1 | 0.13±0.02 |
| BASO(10^9/L) | 0.05±0.03 | 0.03±0.03 | 0.03±0.01 | 0.02±0.01 | 0.03±0.02 | 0.03±0.02 | 0.06±0.02 | 0.04±0.02 |
| NEU(%) | 17.5±6.42 | 14±6.62 | 29.57±3.37* | 29.73±8.73 | 35.37±4.11 | 36.1±11.49 | 15.87±9.6* | 25.07±2.34* |
| LYMPH(%) | 66.33±1.54 | 75.53±2.41* | 61.13±6.42* | 60±5.63 | 57.2±2.07 | 54.97±14.46 | 75.3±9.2 | 68±3.08** |
| MONO(%) | 4.9±1.01 | 4.5±2.12 | 5.27±1.74 | 4.9±0.98 | 4.03±0.71 | 6.37±3.48 | 2.83±1.01 | 3.93±1.38 |
| EO(%) | 10.4±6.95 | 4.83±3.23 | 3.43±1.79 | 4.87±3.66 | 2.87±1.96 | 2.03±1.45 | 4.5±0.26 | 2.2±0.36 |
| BASO(%) | 0.87±0.25 | 1.13±0.45 | 0.6±0.1* | 0.5±0.35 | 0.53±0.21 | 0.53±0.25 | 1.5±0.53 | 0.8±0.4 |
| RBC(10^12/L) | 8.45±0.37 | 8.61±0.6 | 9.15±0.32 | 9.21±0.68 | 8.77±0.58 | 9.3±1.36 | 10.23±1.21 | 9.4±0.42 |
| HGB(g/L) | 147.33±5.51 | 150.33±10.97 | 150.67±6.81 | 154.67±11.59 | 153.33±7.09 | 158.67±18.77 | 176.67±17.04 | 162±4.36 |
| HCT(%) | 44.33±4.19 | 42.43±2.53 | 43.03±2.62 | 44±3.48 | 43.33±0.8 | 45.97±7.31 | 49.53±4.41 | 45.27±1.21 |
| MCV(FL) | 52.4±3.11 | 49.33±0.71 | 47.03±1.61 | 47.73±1.02 | 49.5±2.49 | 49.4±2.7 | 48.6±2.49 | 48.2±0.95 |
| MCH(pg) | 17.43±0.21 | 17.47±0.15 | 16.4±0.56* | 16.8±0.17 | 17.47±0.47 | 17.13±0.58 | 17.3±0.53 | 17.23±0.35 |
| MCHC(g/L) | 334±20.88 | 353.67±7.09 | 349.33±9.29 | 352.33±11.59 | 354±10.58 | 347.33±21.08 | 356.67±8.02 | 358.33±3.51 |
| RDW-CV(%) | 16.53±2.46 | 18.67±6.44 | 17.4±1.44 | 17.5±2.51 | 15.03±1.1 | 19.07±2.84 | 18.3±4.29 | 14.6±1.11 |
| RDW-SD(fl) | 38.57±8.11 | 40.77±15.19 | 36.47±4.06 | 37.47±5.09 | 33.03±2.49 | 42.23±7.83 | 39.97±11.29 | 31.33±3.15 |
| PLT(10^9/L) | 427±127.45 | 324±174.36 | 370.67±121.33 | 300±110.37 | 360±166.95 | 300±94.82 | 436.33±232 | 252.67±67.68 |
| MPV(fl) | 5.73±0.4 | 5.73±0.23 | 5.9±0.75 | 5.53±0.29 | 5.5±0.17 | 6.03±0.49 | 5.5±0.1 | 5.5±0.44 |
| PDW(fl) | 16.37±0.58 | 16.6±0.26 | 16.17±0.23 | 16.13±0.31 | 16.47±0.06 | 16.67±0.29 | 16.67±0.25 | 16.4±0.26 |
| PCT(%) | 0.25±0.08 | 0.19±0.1 | 0.21±0.05 | 0.16±0.05 | 0.2±0.09 | 0.18±0.04 | 0.24±0.12 | 0.14±0.05 |

All data are reported as the mean ± SD. One-way ANOVA followed by Games-Howell *post hoc* test, compared with Control, ^*^*P*<0.05, ^**^*P*<0.01. WBC, white blood cells; NETU, neutrophils; LYMPH, lymphocytes; MONO, monocytes; EO, eosinophil; BASO, basophils; NEU, neutrophils; RBC, red blood cell; HGB, hemoglobin; HCT, hematocrit; MCV, mean corpuscular volume; MCH, melanin-concentrating hormone; MCHC, mean corpuscular hemoglobin concentration; RDW-CV, red cell distribution width- coefficient of variation; RDW-SD, red cell distribution width- standard deviation; PLT, platelet; MPV, mean platelet volume; PDW, platelet distribution width; PCT, procalcitonin.

Table S8 Effect of daily oral administration of jaranol on liver and kidney function in mice

| Parameters | Male (n=3) | | | | Female (n=3) | | | |
| --- | --- | --- | --- | --- | --- | --- | --- | --- |
|  | control | 50mg/kg/day | 100mg/kg/day | 200mg/kg/day | control | 50mg/kg/day | 100mg/kg/day | 200mg/kg/day |
| ALT(U/L) | 25.87±1.8 | 40.76±22.15 | 21.02±3.3 | 17.18±2.65^**^ | 28.79±7.42 | 24.37±9.7 | 21.66±5.94 | 19.85±3.42 |
| AST(U/L) | 84.38±17.35 | 205.35±112.88 | 72.37±6.5 | 68.82±15.28 | 86.08±10.91 | 111.15±38.91 | 85.91±22.86 | 75.86±15.93 |
| T-Bil(μmol/L) | 13.78±3.08 | 10.1±4.78 | 15.84±0.59 | 15.8±4.12 | 12.42±1.25 | 12.76±0.67 | 11.03±1.85 | 14.24±2.24 |
| Cre(μmol/L) | 17±2.9 | 28.79±2.98^*^ | 17.49±2.52 | 20.18±1.22 | 20.65±1.6 | 23.88±2.08 | 20.29±4.37 | 19.38±1.54 |

All data are reported as the mean ± SD. One-way ANOVA followed by Games-Howell *post hoc* test, compared with Control, ^*^*P*<0.05, ^**^*P*<0.01. ALT, alanine aminotransferase; AST, aspartate aminotransferase; T-Bil, total bilirubin; Cre, Creatinine.

Table S9 Effect of daily oral administration of jaranol on lipid profile in mice

| Parameters | Male (n=3) | | | | Female (n=3) | | | |
| --- | --- | --- | --- | --- | --- | --- | --- | --- |
|  | control | 50mg/kg/day | 100mg/kg/day | 200mg/kg/day | control | 50mg/kg/day | 100mg/kg/day | 200mg/kg/day |
| LDL-C(mmol/L) | 1.63±0.39 | 1.44±0.13 | 1.29±0.13 | 1.53±0.08 | 1.39±0.1 | 2.09±0.52 | 1.23±0.1 | 1.27±0.23 |
| HDL-C(mmol/L) | 2.14±0.44 | 1.68±0.37 | 2.31±0.3 | 3.08±0.33 | 1.9±0.17 | 2.91±0.3^**^ | 1.89±0.27 | 1.81±0.29 |
| CHO(mmol/L) | 2.81±0.45 | 2.29±0.37 | 2.5±0.32 | 3.08±0.17 | 2.39±0.2 | 3.68±0.64 | 2.19±0.24 | 2.15±0.3 |

All data are reported as the mean ± SD. LDL-C, low-density lipoprotein cholesterol; HDL-C, high-density lipoprotein cholesterol; CHO, cholesterol. ANOVA one way followed by Games-Howell *post hoc* test, compared with Control, ^**^*P*<0.01.

Table S10 Histopatholohical studies were scored by pathologist

| Organ | Expert staining score |
| --- | --- |
| heart | 4.5±1.38 |
| liver | 4±0.89 |
| spleen | 4.17±0.98 |
| lung | 4±0.89 |
| kidney | 4.33±0.82 |

All data are reported as the mean ± SD. for n=6 per group.
